# Influence of Decomposition Dynamics of Plant–Animal Organic Matter on Distribution Patterns of Calyptrate Flies

**DOI:** 10.3390/insects17080771

**Published:** 2026-07-27

**Authors:** Weiqing Zheng, Yinuo Yan, Jingzhi Huang, Siping Li, Qianfeng Xia

**Affiliations:** NHC Key Laboratory of Tropical Disease Control, School of Life Sciences and Medical Technology, Hainan Medical University, Haikou 571199, China

**Keywords:** plant tissues, animal tissues, decomposition dynamics, fly assemblages, distribution pattern

## Abstract

Understanding how decomposing organic matter attracts calyptrate flies is fundamental to both medical entomology and forensic science, yet the differential effects of substrate type and decomposition stage remain poorly characterized in tropical environments. In this study, we established controlled decomposition gradients for fish viscera (animal tissue) and leafy vegetables (plant tissue) at 37 °C and 65% RH, defining six physicochemical stages per substrate based on color, odor, and ammonia emissions. Over 20 days, in Haikou, Hainan, we deployed 30 spatially replicated traps per stage, collecting 1538 specimens spanning 13 species across four families. Animal substrates attracted 6.8-fold higher fly densities than plant materials (1.48 vs. 0.22 flies/cage/hour; *p* < 0.01), with *Limnophora nudiseta* dominating animal-baited traps (68.43%) and *Anthomyia illocata* prevailing in plant traps (43.94%). Decomposition stage critically modulated attraction: mid–late decay stages (2–5) yielded 3.6-fold more flies on animal tissues than fresh stages, accompanied by a successional shift from *A. illocata* to *L. nudiseta* as the primary colonizer. Plant tissues showed incremental but non-significant density increases, and were consistently dominated by *A. illocata*. These findings demonstrate that substrate identity and decomposition dynamics jointly govern fly abundance, community composition, and niche partitioning. Our mechanistic framework offers practical guidelines for optimizing fly surveillance through species-specific bait selection and decay-stage calibration, with direct applications in vector control, public health monitoring, and forensic post mortem interval estimation.

## Simple Summary

Flies are more than just a nuisance—they can carry harmful germs from rotting waste to human food. To better understand what attracts them, we placed two types of rotting materials—fish guts (animal-based) and leafy vegetables (plant-based)—in traps, and counted which fly species were drawn to each. Over 20 days in Hainan, China, we collected over 1500 flies representing 13 different species. We found that rotting fish guts attracted far more flies than rotting vegetables, and the types of flies present changed as the materials decayed further. For example, one fly species preferred fresher fish, while another took over as the fish became more decomposed. These findings help us predict where flies are likely to gather and breed. This is useful for designing better fly control programs, and may even aid forensic investigations by helping to estimate how long a body has been decomposing.

## 1. Introduction

China has documented 1547 described calyptrate fly species classified under nine families [1]. Surveillance data obtained in 2019 revealed 14 predominant calyptrate species: *Musca domestica* (house fly), *Lucilia sericata* (common green bottle fly), *Muscina stabulans* (false stable fly), *Chrysomya megacephala* (oriental latrine fly), *Musca sorbens* (bazaar fly), *Lucilia cuprina* (Australian sheep blowfly), *Lucilia illustris* (lustrous green bottle fly), *Aldrichina grahami* (forest blow fly), *Fannia prisca*, *Fannia canicularis* (lesser house fly), *Protophormia terraenovae* (northern blowfly), *Calliphora vicina* (blue bottle fly), *Phormia regina* (black blowfly), with additional unidentified sarcophagids. *Musca domestica* constituted the dominant species (27.59% of total captures), followed by *L. sericata* (17.54%) [2]. Among the predominant calyptrate fly species, *L. sericata* has a known preference for decaying animal materials, while *M. domestica* is a generalist that exploits a wide range of organic matter, including decaying plant and animal materials [3,4].

Calyptrate flies inhabit areas that overlap with human living and working environments, where their dense and diverse populations pose ongoing threats to public health and the goals of the Healthy China initiative. A key behavioral feature, simultaneous feeding and defecation, enables these flies to mechanically transfer enteric pathogens from waste-breeding sites to human food sources. This direct contamination not only compromises food hygiene and causes aesthetic nuisance but also facilitates the transmission of diarrheal diseases. Surveillance studies confirm that flies transmit multiple diarrheal disease agents, including *Salmonella enterica*, *Shigella* spp., *Campylobacter* spp., and *Escherichia coli* O157:H7 [5,6]. More critically, flies can also act as vectors for antimicrobial-resistant bacteria and emerging pathogens, thereby compounding the already serious threats to disease control and food safety [7,8]. Fly-borne disease outbreaks disproportionately burden developing regions such as rural areas of Lebanon, sub-Saharan Africa, and Pakistan. Notably, however, developed nations remain vulnerable, as exemplified by Japan’s seasonal *Yersinia enterocolitica* gastroenteritis zoonosis outbreaks during summer [6].

Human outdoor activities modulate fly involvement in organic decomposition processes, prompting investigation into reciprocal effects of biodegradative succession on fly distribution patterns [9]. Calyptrate flies primarily forage on decomposing organic matter, especially food waste and spoiled products from wet markets (e.g., decaying vegetables, raw fish viscera). That is because flies are strongly attracted by the volatile organic compounds (VOCs) like trimethylamine, ammonia, indole, and linoleic acid which are emitted from the decomposing matter [10]. Hainan province’s year-round hyperthermal-humid climate (mean annual RH > 80%, T > 24 °C) accelerates organic decomposition, which establishes optimal fly breeding habitats. Crucially, physicochemical transitions in organic matters manifest chromatic alterations, VOC profile shifts, and texture metamorphosis during decomposition stages, and these may significantly alter olfactory attractiveness to flies. Mechanistically, these changes are driven by microbial succession. Proteolysis and amino acid decarboxylation generate characteristic VOCs (e.g., indole, skatole, and cadaverine) that act as long-range olfactory attractants, while lipid peroxidation and Maillard reactions induce color shifts from bright to dark brown, providing medium-range visual cues. Concurrently, enzymatic maceration softens substrate tissues, which facilitates larval feeding and oviposition site selection. However, few studies in tropical China have evaluated how the calyptrate fly community changes within organic decomposition processes. This study aimed to characterize the distribution patterns of flies on decomposing plant versus animal substrates across decomposition stages, and to determine whether these patterns differ significantly between the two organic matter types. Results should serve as mechanistic groundwork for fly density surveillance optimization and for fly foraging ecology modeling.

## 2. Material and Methods

### 2.1. Preparation of Decayed Baits and Setup of the Experimental Groups

Oviposition and larval development substrates for calyptrate flies in urban settings are predominantly in domestic organic refuse, which can be broadly classified into vegetal and animal matter. The vegetal fraction is largely represented by desiccated, chlorotic leaf vegetables, while the animal fraction is primarily comprised of abattoir by-products: notably, viscera and other internal organs. In this study, yellow leafy vegetables and the viscera of marine fish commonly consumed in Hainan were used as plant- and animal-derived baits for flies. Based on preliminary observations, the decomposition process of the plant material (yellow leafy vegetables) was shorter under conditions of 37 °C and 65% relative humidity, whereas that of the fish viscera was relatively longer. Accordingly, the yellow leafy vegetables were divided into five portions and placed simultaneously in an artificial climate chamber (Shanghai Boxun Industrial Co., Ltd., Shanghai, China) set at 37 °C and 65% relative humidity. During this period, one portion was taken out every 4 h, and the corresponding decomposition stage was recorded, along with changes in appearance (including color, texture and shape) and odor (Table 1). For the fish viscera, examinations were performed every 12 h, and all other procedures were the same as those described for the yellow leafy vegetables. Yellow leafy vegetables and viscera of marine fish before decay were set up as controls.

### 2.2. Determining Fly Infestation in Plant- and Animal-Tissue-Baited Traps

#### 2.2.1. Collection Site

The survey was conducted on the island of Hainan, the second largest island in China. The outstanding feature of this island is its climatic variety, ranging from very humid rain forest in the east to arid land in the west. The other notable feature of Hainan is its topographic variety, as it features an elevated central massif surrounded by low-lying peripheral terrain. The average annual temperature is 22.5–25.6 °C. This study was carried out in the Chengxi campus of Hainan Medical University in Haikou, Hainan. During the 20-day monitoring period, temperatures ranged between 22 °C and 33 °C, with clear weather, wind force 1–2 (Beaufort scale), and relative humidity of 63–75%.

#### 2.2.2. Collection Technique

**Figure 1:**
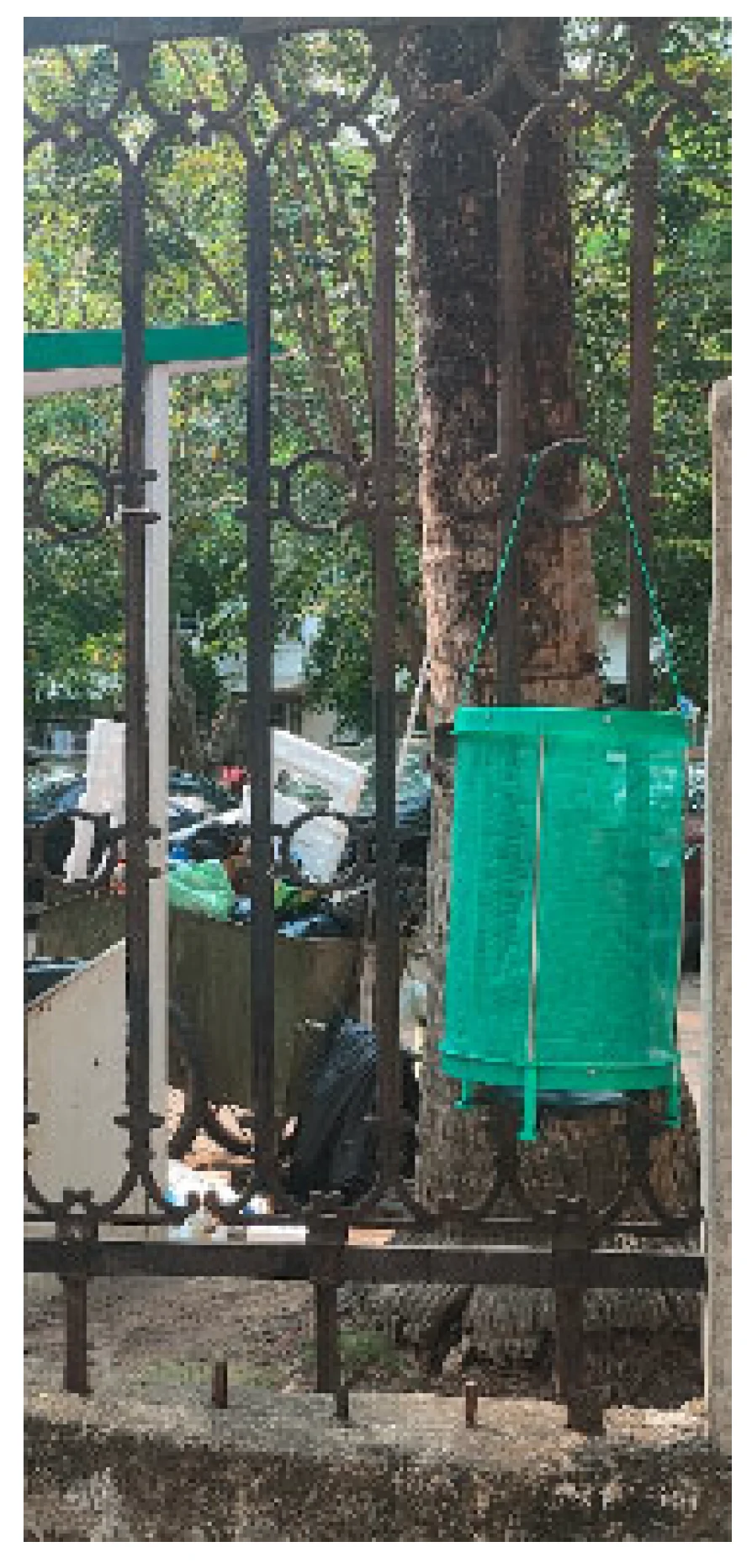
Fly trap set up in a monitoring site.

The monitoring method was in accordance with the National Standard of the People’s Republic of China (GB/T 23796-2009) [11]. In brief, trapping devices were suspended and positioned in well-ventilated locations, avoiding direct sunlight so that operational stability and consistent trapping efficacy were maintained (Figure 1). Fly traps baited with different materials and decomposition stages were randomly placed at monitoring sites, spaced 20 m apart, and periodically rotated among sites to minimize the influence of site position on fly distribution. Trap cage elevation was regulated at 0.1–0.3 m above ground level, corresponding to flies’ typical flight altitude and activity range. Trapping operations were conducted daily from 0900–1500 h (UTC+8), constituting a contiguous six-hour sampling period per diurnal cycle. Animal/plant tissues (50–60 g) in varying stages of decomposition were placed in bait trays, with 30 spatially distributed replicates per treatment. Unspoiled specimens served as experimental controls. When the traps were collected, the bag with flies was sealed and fetched, and then taken back to the laboratory of Hainan Medical University in Haikou, Hainan. For further identification, the files were frozen to death at −20 °C for at least three hours.

### 2.3. Identification

Adult identifications were made under a microscope (Olympus Corporation, Tokyo, Japan) by an entomologist on the basis on morphological characteristics, using the keys of Fan, and Zhou and Chu [1,12]. Voucher specimens were deposited at Hainan Medical University, located at Chengxi Campus, Haikou, Hainan.

### 2.4. Data Analysis

Fly communities were analyzed both by bait material and decomposition stage. We calculated relative abundance, species richness, and species composition for each decomposition stage based on the bait material, either plant or animal tissue. Relative abundances were not normally distributed in plant or animal tissue at different decomposition stages; these were therefore analyzed with Kruskal–Wallis one-way ANOVA to determine regional and seasonal differences in the relative abundances of the six decomposition stages. The analysis was followed by a Wilcoxon rank-sum test when Kruskal–Wallis results were statistically significant (*p* < 0.05). All statistical calculations were performed using GraphPad Prism software (version 10).

## 3. Results

### 3.1. Richness and Relative Abundance of Species at Sampling Site

**Figure 2:**
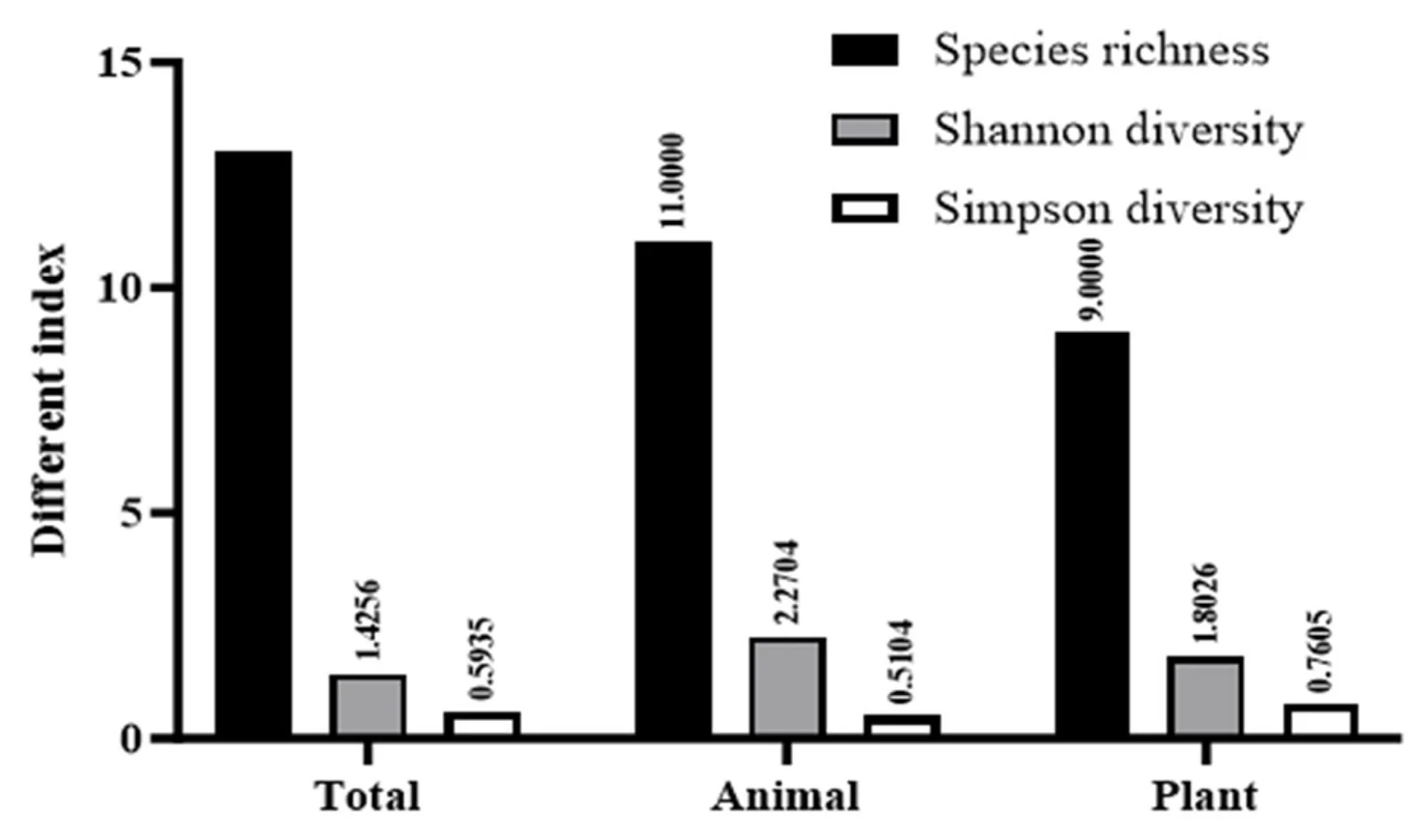
Diversity of fly species captured in Haikou. Species richness is estimated as the number of observed species. Shannon diversity (H′) is calculated as $H^{\prime }=-\sum p_{i}ln\;p_{i}$, and Simpson diversity is computed as $D=1-\sum p_{i}^{2}$, where $p_{i}$ represents the relative abundance of the i-th species.

A total of 1538 specimens were collected during the fly surveillance. A high level of alpha diversity was observed at the monitoring site, as indicated by a species richness of 13, a Shannon index of 1.4256, and a Simpson index of 0.5935 (Figure 2). Muscidae comprised six species: *Musca domestica*, *Musca sorbens*, *Fannia prisca*, *Limnophora nudiseta*, *Atherigona orientalis*, and *Hydrotaea chalcogaster*. Sarcophagidae included three species: *Boettcherisca peregrina*, *Parasarcophaga dux*, and *Parasarcophaga ruficornis*. Calliphoridae contained three species: *Hemipyrellia ligurriens*, *Chrysomya megacephala*, and *Lucilia sericata*. Anthomyiidae was represented by *Anthomyia illocata*. *Limnophora nudiseta* demonstrated an absolute predominance, with a 61.32% species composition ratio (SCR), significantly exceeding the second-ranked *A. illocata* (SCR = 13.26%) and the third-ranked *P. dux* (SCR = 10.01%). Minor species included *F. prisca* (SCR = 0.59%) and *H. chalcogaster* (SCR = 0.91%) (Table 2).

### 3.2. Differences in Fly Distribution Pattern Between Plant and Animal Tissue-Baited Traps

**Figure 3:**
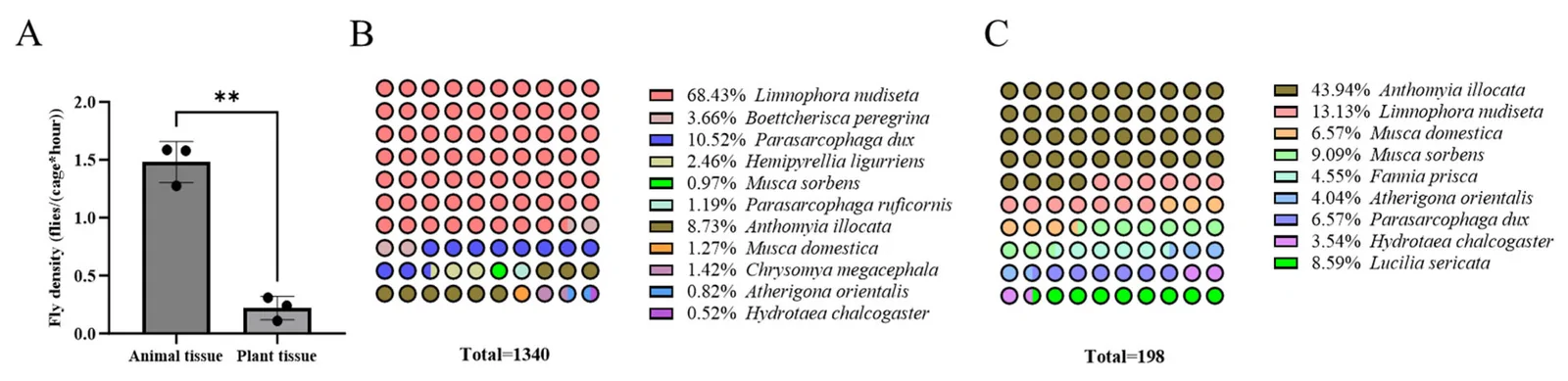
Fly distribution pattern in animal-tissue-baited traps versus plant-tissue-baited traps. (**A**) fly density on animal and plant tissues. (**B**) Composition of fly assemblages trapped in animal tissues. (**C**) Composition of fly assemblages trapped in plant tissues. ** *p* < 0.01.

Over the entire trapping period of 20 d, the density of flies lured onto animal tissue was 1.48 flies/(cage·hour) (95% confidence interval (CI), 1.28–1.68 flies/(cage·hour)), significantly higher than for flies (0.22, 95%CI (0.11–0.33) flies/(cage·hour)) captured on plant tissue (t = 10.70, df = 3.19, *p* = 0.00l3) (Figure 3A). Animal-based baits attracted 1340 flies comprising 11 fly species, with a Shannon diversity index of 2.2724 and a Simpson diversity index of 0.5104 (Figure 2). *Limnophora nudiseta* represented the predominant taxon (68.43% relative abundance), with *Parasarcophaga dux* (10.52%) and *Anthomyia illocata* (8.73%) ranked second and third, respectively. In comparison, plant-based baits captured 198 flies across 9 species, yielding a Shannon index of 1.8026 and a Simpson index of 0.7605 (Figure 2). *Anthomyia illocata* constituted the dominant species (43.94% relative abundance), while *L. nudiseta* accounted for 13.13%. These results indicate that while animal-derived baits attracted a greater richness of species, they exhibited stronger dominance (i.e., lower evenness) than plant-derived baits, which supported a more equitable distribution of individuals among species (Figure 3B,C).

### 3.3. Effect of Duration of Decomposition of Plant and Animal Tissues on Fly Distribution

**Figure 4:**
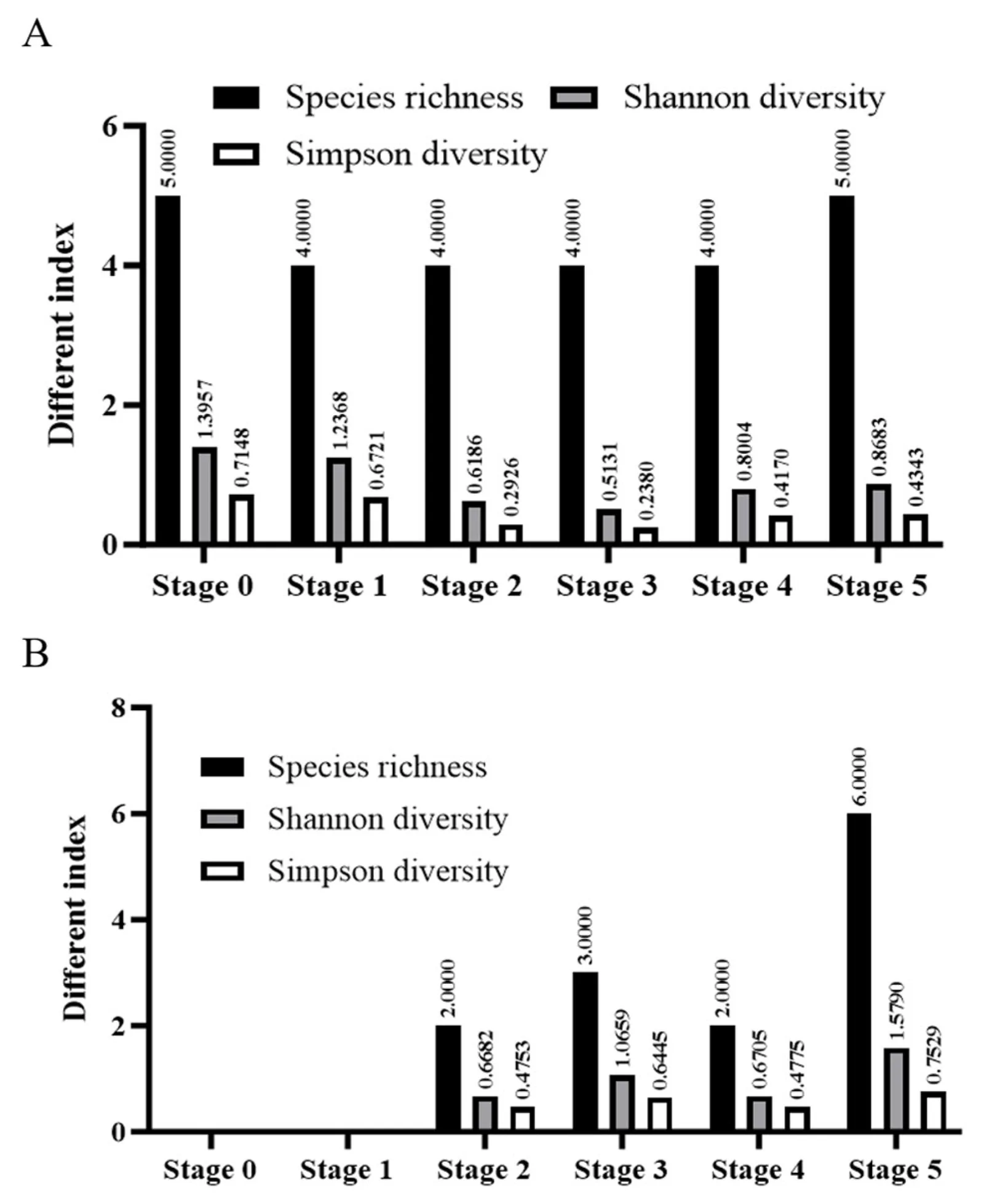
Diversity of fly species captured in animal and plant tissues. (**A**) Diversity of fly species captured in animal tissues. (**B**) Diversity of fly species captured in plant tissues.

**Figure 5:**
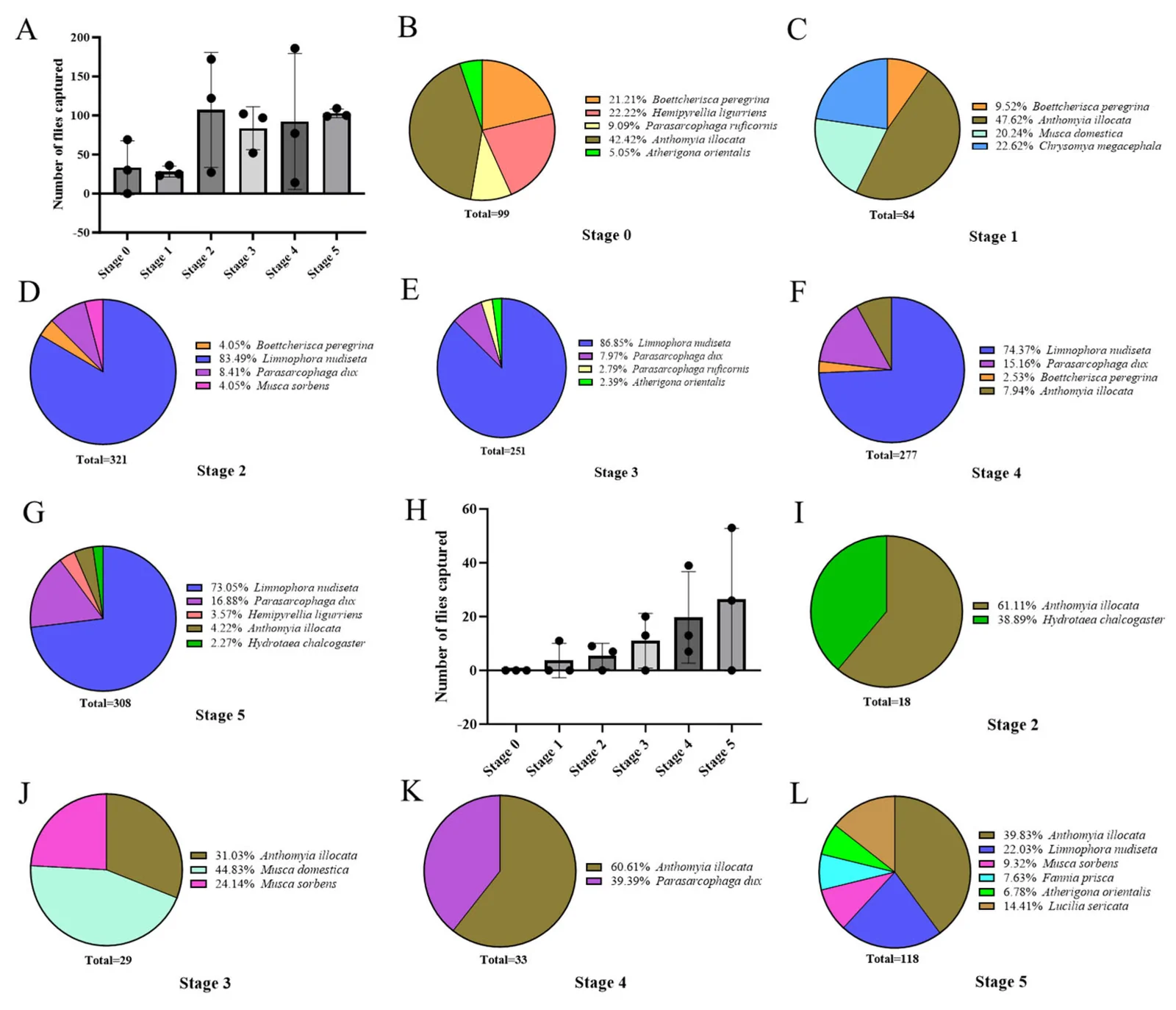
The state of decomposition in animal and plant tissues related to fly distribution. (**A**) Numbers of flies captured in animal tissues in 30 cages at stages 0 to 5. (**B**–**G**) Composition of fly assemblages trapped in animal tissues at stages 0 to 5. (**H**) Numbers of flies captured in plant tissues in 30 cages at stages 0 to 5. (**I**–**L**) Composition of fly assemblages trapped in plant tissues at stages 2 to 5.

The state of decomposition of animal tissues significantly influenced fly capture dynamics. Fly diversity exhibited a distinct trough-then-peak pattern across decomposition stages: species richness declined from 5 at stage 0 to 4 at stages 1–4, then rebounded to 5 at stage 5. The Shannon diversity index decreased from 1.3957 at stage 0 to a minimum of 0.5131 at stage 3, followed by an increase to 0.8683 at stage 5. Similarly, the Simpson diversity index dropped from 0.7148 at stage 0 to 0.2380 at stage 3, then rose to 0.4343 at stage 5 (Figure 4). During early decomposition (stages 0–1), mean daily trap yields were low (28–33 individuals), with *A. illocata* predominating. In mid–late decomposition phases (stages 2–5), capture abundance increased (84–107 individuals/day) but showed no statistically significant difference versus the early phase (*p* = 0.2096). *Limnophora nudiseta* became the dominant taxon during this period (Figure 5A–G). In the case of plant tissues, baits showed a more complex trajectory, exhibiting a rise-then-decline-then-rise pattern: species richness increased from 0 at stage 0 to 3 at stage 3, decreased to 2 at stage 4, and finally climbed to 6 at stage 5. The Shannon index followed a parallel trend, rising from 0 at stage 0 to 1.0659 at stage 3, declining to 0.6705 at stage 4, and increasing again to 1.5790 at stage 5. The Simpson index showed analogous fluctuations, ascending from 0 at stage 0 to 0.6445 at stage 3, falling to 0.4775 at stage 4, and rebounding to 0.7529 at stage 5 (Figure 4). Fly captures exhibited temporal escalation from 0 individuals/day in fresh tissues to 26.33 individuals/day at stage 5 decomposition. However, no significant differences emerged among decomposition stages (*p* = 0.2201). *Anthomyia illocata* constituted the dominant population across all decomposition stages except stage 3 (Figure 5H–L).

## 4. Discussion

The observed density of flies in monitoring programs depends on multiple interacting factors, such as the sampling technique employed, the type of bait used, the availability and quality of breeding sites, local weather patterns, and the time of year [13,14,15,16]. In the current study, 1538 synanthropic fly specimens representing 13 species across 11 genera were taxonomically verified, with *L. nudiseta* (61.31%) and *A. illocata* (13.26%) being identified as dominant taxa, while *F. prisca* (0.59%) and *H. chalcogaster* (0.91%) constituted rare species. Divergent community patterns emerged when our findings were contextualized with the two prior regional surveys. Qu (2024) isolated 32 species from 16 genera, establishing *C. megacephala* and *B. peregrina* as dominants across Hainan’s forested ecosystems, whereas Wang et al. (2020)’s national surveillance program documented *Lucilia cuprina* prevalence in typical urban interfaces including market and residential transects [2,17]. These significant variations in diversity, abundance, and species composition are fundamentally attributed to sampling differences. Our study confined sampling within an oligotrophic campus matrix (academic buildings/canteens). This contrasts with Qu’s wilderness-focused collection along highways/mountain corridors and Wang’s anthropogenically enriched urban ecotone sampling. Such habitat stratification drives distinct assembly rules within calyptrate fly guilds. In the present study, despite a limited sampling area we still documented 13 species, including the rarely observed *A. orientalis*.

Raw fish viscera provide protein-rich matrices enriched with enzymes, lipids, amino acids, hydroxyapatite, collagen, and gelatin. Their decomposition generates putrescine, hydrogen sulfide, and indole derivatives [18,19], imparting intensely malodorous characteristics. Conversely, fresh vegetable foliage contains RuBisCO (Ribulose-1,5-bisphosphate carboxylase/oxygenase) as the primary protein constituent alongside substantial cellulose with trace minerals, vitamins, and folate [20]. Plant tissues at early–mid decomposition stages emit detectable alcoholic fermentation odors, progressing to ammoniacal and ultimately putrid smells in terminal phases. This chemical disparity might govern differential attractiveness across distinct decomposition substrates. Animal substrates and their decomposed products seemed consistently to attract significantly higher calyptrate fly abundances, with altered community structures, compared to plant materials. *Limnophora nudiseta* demonstrated a pronounced preference for decomposing animal tissues, numerically dominating these baits by substantial margins. In stark contrast, *A. illocata* was predominant on vegetal decomposition substrates, exhibiting an approximately 3.0-fold greater density than *L. nudiseta*. This reflects species-specific breeding resource selection, a behavioral adaptation driving niche partitioning within calyptrate fly assemblages.

Throughout the decomposition process, both fly diversity and abundance exhibited a general pattern of initial decline followed by a subsequent increase. This non-monotonic trend is likely attributable to differences in volatile emission profiles among fresh, moderately decomposed, and highly decomposed animal and plant tissues. In fresh specimens, volatiles are primarily derived from the intrinsic biochemical composition of the tissues themselves. As decomposition progresses, microbial activity intensifies, leading to the production of a wide array of novel volatile compounds. Consequently, the composition of the attracted fly assemblage shifts predictably across different decomposition stages, reflecting the changing chemical landscape of the bait. For example, at the early decomposition stage animal substrates captured mostly *A. illocata* flies, then changed to mainly attract *L. nudiseta* at mid–late decomposition stages. Future fly surveillance and biological trait investigations should therefore employ species-specific substrate selection with precise decomposition-state calibration to optimize catch rates of target taxa. This action will provide critical methodological frameworks for medical entomology and forensic applications. To illustrate, in discarded fish viscera, a predominance of *A. illocata* suggests that the substrate remains relatively fresh or is in the early stages of decomposition. Conversely, an abundance of *L. nudiseta* indicates that the substrate has undergone a longer period of putrefaction. By integrating these entomological indicators with ambient temperature and relative humidity data, a reasonable estimation of the time since fish slaughter can be made. These findings have significant implications for forensic entomology, particularly in estimating the post mortem interval (PMI) from decomposing remains.

Several limitations are present in the current study. Work still to be done includes quantification of dominant VOCs from decomposing biomass, as well as compositional analysis of post-decay matrices. Furthermore, assessment of the attractant efficacy of individual compounds or synergistic mixtures across calyptrate fly species lies beyond this study’s scope. These investigations constitute separate research initiatives currently underway.

## 5. Conclusions

This study (carried out in Hainan, China) establishes that organic substrate variability and decomposition dynamics collectively regulate calyptrate fly capture abundance, population density, and community structure. These findings hold significant implications for fly monitoring standardization and calyptrate fly biology research. Moreover, our data reveal unique calyptrate fly distribution patterns in localized tropical habitats.

## Figures and Tables

**Table 1:** Putrefaction dynamics of plant greens and animal tissues.

Bait Type	Decomposition Stage	Qualitative Color	Objective Color (CIELAB *L*ab) *	Qualitative Odor	Objective Odor (NH_3_, ppm) ^†^
Greens	Initial (Stages 0–1)	Vibrant green	*L*: 48 ± 3, a: –14 ± 2, b: 24 ± 2	Vegetable aroma	<2
	Midterm (Stages 2–3)	Dark olive	*L*: 35 ± 4, a: –5 ± 3, b: 18 ± 3	Slight ethanol-fermentation aroma	5–15
	Late (Stages 4–5)	Browning	*L*: 28 ± 5, a: 6 ± 4, b: 12 ± 4	Ammonia-like putridity	>30
Animal tissues	Initial (Stages 0–1)	Dark red	*L*: 32 ± 4, a: 18 ± 3, b: 8 ± 2	Fishy odor	3–8
	Midterm (Stages 2–3)	Purplish discoloration	*L*: 28 ± 5, a: 12 ± 4, b: 6 ± 3	Mild putrescence	20–40
	Late (Stages 4–5)	Clay-colored appearance	L: 25 ± 6, a: 5 ± 4, b: 10 ± 5	Pronounced foulness	>60

* CIELAB values measured with a Minolta colorimeter (illuminant D65, 10° observer) on three randomly selected spots per sample. L represents lightness (brightness), ranging from 0 (black) to 100 (white). “a” represents the red–green axis. Positive values (“+a”) indicate red, while negative values (“−a”) indicate green. “b” represents the yellow–blue axis. Positive values (“+b”) indicate yellow, while negative values (“−b”) indicate blue. ^†^ Ammonia concentration measured by an electrochemical sensor (GasAlert Extreme) at 5 cm above the sample surface.

**Table 2:** Total numbers and percentages of fly species collected in Haikou in March and April 2025.

Family	Genus	Species	Total Collected	Percent (%)
Muscidae	*Musca*	*domestica*	30	1.95
		*sorbens*	31	2.02
	*Fannia*	*prisca*	9	0.59
	*Limnophora*	*nudiseta*	943	61.32
	*Atherigona*	*orientalis*	19	1.24
	*Hydrotaea*	*chalcogaster*	14	0.91
Sarcophagidae	*Boettcherisca*	*peregrina*	49	3.19
	*Parasarcophaga*	*dux*	154	10.01
		*ruficornis*	16	1.04
Calliphoridae	*Hemipyrellia*	*ligurriens*	33	2.15
	*Chrysomya*	*megacephala*	19	1.24
	*Lucilia*	*sericata*	17	1.11
Anthomyiidae	*Anthomyia*	*illocata*	204	13.26

## Data Availability

Data pertinent to this study have been shown in conjunction with the present work.

## References

[1] Fan Z.D. (1992). Key to the Common Flies of China.

[2] Wang X.S., Wu H.X., Liu Q.Y. (2020). National surveillance report on flies in China, 2019. Chin. J. Vector Biol. Control.

[3] Keiding J., World Health Organization (1986). The House-Fly: Biology and Control.

[4] Huntington T.E., Higley L.G. (2010). Decomposed flesh as a vitellogenic protein source for the forensically important *Lucilia sericata* (Diptera: Calliphoridae). J. Med. Entomol..

[5] Shahanaz E., Zwally K.M., Powers C., Lyons B., Kaufman P., Athrey G., Taylor T.M. (2025). Flies as vectors of foodborne pathogens through food animal production: Factors affecting pathogen and antimicrobial resistance transmission. J. Food Prot..

[6] Graczyk T.K., Knight R., Gilman R.H., Cranfield M.R. (2001). The role of non-biting flies in the epidemiology of human infectious diseases. Microbes Infect..

[7] Stoops C.A., Smith H.J., Pitzer J.B., Milan N.F., Nayduch D. (2026). Antimicrobial Resistance and Insecticide Resistance Co-Occurrence in Synanthropic Flies and Their Implications for Military Health in Large-Scale Combat Operations.

[8] AbdAllah O.R., Gabre R.M., Mohammed S.A., Korayem A.M., Hussein H.E., Ahmad A.A. (2025). Evaluating the role of synanthropic filth flies in the transmission of zoonotic parasites: Field and laboratory evidence from different animal rearing sites in upper Egypt with focus on *Cryptosporidium* spp. BMC Vet. Res..

[9] Dufek M.I., Battan-Horenstein M., Larrea D.D., Mulieri P.R. (2025). Decomposition dynamics: The influence of anthropogenic disturbance on organic matter degradation by sarcosaprophagous flies. J. Med. Entomol..

[10] Baleba S. (2026). Advances and Prospects in Odour-based Management of Filth Flies. J. Chem. Ecol..

[11] (2009). Surveillance Methods for Vector Density.

[12] Zhou M.H., Chu H.L. (2019). Hand Book for Classification and Identification of Main Vectors.

[13] Olea M.S., Patitucci L.D., Mariluis J.C., Alderete M., Mulieri P.R. (2017). Assessment of Sampling Methods for Sarcosaprophagous Species and Other Guilds of Calyptratae (Diptera) in Temperate Forests of Southern South America. J. Med. Entomol..

[14] Mulieri P.R., Patitucci L.D., Olea M.S. (2015). Sex-biased Patterns of Saprophagous Calyptratae (Diptera) Collected with Different Baits of Animal Origin. J. Med. Entomol..

[15] Vasconcelos S.D., Barbosa T.M., Oliveira T.P.B. (2015). Diversity of forensically-important dipteran species in different environments in northeastern Brazil, with notes on the attractiveness of animal baits. Fla. Entomol..

[16] Michaud J.-P., Schoenly K.G., Moreau G. (2012). Sampling flies or sampling flaws? Experimental design and inference strength in forensic entomology. J. Med. Entomol..

[17] Qu Y.H. (2024). Investigation and Establishment of Regional Genebanks of Necrophilic Flies and Application of Multi-Method Species Identification in Forensic Practice in Hainan. Ph.D. Thesis.

[18] Estiasih T., Ahmadi K., Ali D., Nisa F., Suseno S., Lestari L. (2021). Valorisation of viscera from fish processing for food industry utilizations. IOP Conference Series: Earth and Environmental Science.

[19] Qu Z.Y. (2017). Sanitary Microbiology.

[20] Thakur V., Mal D., Soga K., Gandhi A. (2022). A review on nutritional quality of green leafy vegetables. Ecol. Environ. Conserv..

